# Reconstitution of Polyketide-Derived Meroterpenoid Biosynthetic Pathway in *Aspergillus oryzae*

**DOI:** 10.3390/jof7060486

**Published:** 2021-06-16

**Authors:** Takayoshi Awakawa, Ikuro Abe

**Affiliations:** 1 Laboratory of Natural Products Chemistry, Graduate School of Pharmaceutical Sciences, The University of Tokyo, Bunkyo-ku, Tokyo 113-0033, Japan; 2 Collaborative Research Institute for Innovative Microbiology, The University of Tokyo, Yayoi 1-1-1, Bunkyo-ku, Tokyo 113-8657, Japan

**Keywords:** *Aspergillus oryzae*, heterologous expression, secondary metabolites, meroterpenoid

## Abstract

The heterologous gene expression system with *Aspergillus oryzae* as the host is an effective method to investigate fungal secondary metabolite biosynthetic pathways for reconstruction to produce un-natural molecules due to its high productivity and genetic tractability. In this review, we focus on biosynthetic studies of fungal polyketide-derived meroterpenoids, a group of bioactive natural products, by means of the *A. oryzae* heterologous expression system. The heterologous expression methods and the biosynthetic reactions are described in detail for future prospects to create un-natural molecules via biosynthetic re-design.

## 1. Introduction

*Aspergillus oryzae* is a fungus that has been utilized for over 2000 years in the Japanese fermentation industry to yield sake, miso, and soy sauce, and for industrial enzyme production. *A. oryzae* is an important heterologous expression host for biosynthetic genes from *Aspergillus* species that produce numerous secondary metabolites, such as mycotoxins, aflatoxin, medicinal compounds, lovastatin, and pigments such as emodin [1,2,3], as well as other secondary metabolite-producing fungi. For investigations on their biosynthesis, the heterologous expression system is a valuable tool, as it provides information about their reactions based on the products accumulated in the transformants. The *A. oryzae* genome includes some genes encoding secondary metabolite biosynthetic enzymes, such as the non-ribosomal peptide synthetase, which is responsible for the production of toxic compounds, such as cyclopiazonic acid. However, most of these genes, including cyclopiazonic acid synthetases, are dysfunctional or not expressed in the *A. oryzae* strains used in fermented food, and many have been recognized as safe strains that do not produce any toxic compounds [4]. In addition, *A. oryzae* possesses a strong metabolic flux to supply precursors of polyketides, terpenoids, and peptides, as judged by the high yields of heterologous expression products. Furthermore, genetic manipulation methods, such as gene deletion or heterologous expression, strong promoters (e.g., PamyB and PenoA) and multicopy vectors [5,6], and the genetically tractable mutated strains *A. oryzae* M-2-3 and NSAR1, have been established [7,8]. The long-standing efforts of academic and industrial investigators have made this strain an excellent chassis for the heterologous expression of secondary metabolite genes. By using this strain, we can easily access new biosynthetic pathways and construct systems for the production of beneficial compounds.

**Figure 1:**
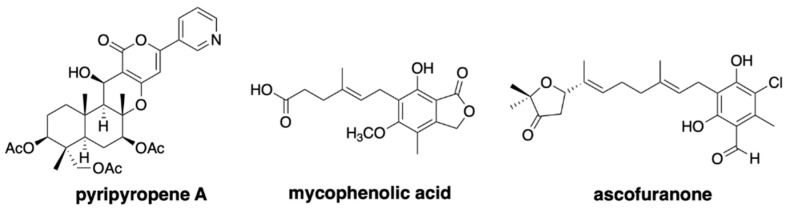
Representative bioactive fungal meroterpenoids.

**Figure 2:**
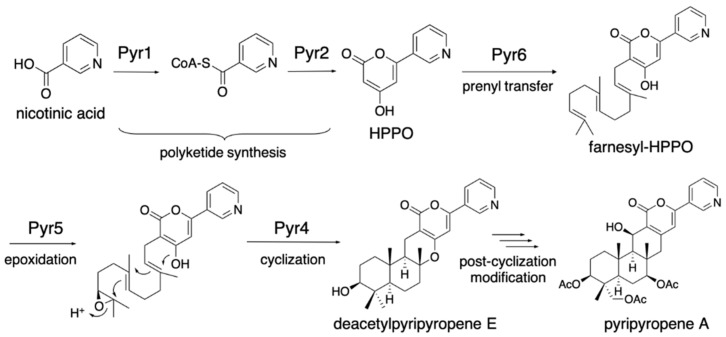
Biosynthetic pathway of pyripyropene A.

The fungal meroterpenoids are one of the groups of natural products that include various medicinally important compounds, such as the acyl-CoA:cholesterol acyltransferase inhibitor pyripyropene, the immunosuppressant mycophenolic acid, and the anti-trypanosomiasis ascofuranone (Figure 1) [9]. The biosynthesis of fungal meroterpenoids can be separated into five parts: polyketide synthesis, prenyl transfer, epoxidation, cyclization, and post-cyclization modification [10,11,12], as exemplified by pyripyropene A biosynthesis (Figure 2). By using the versatile *A. oryzae* host, which synthesizes polyketide and terpenoid compounds with high yields, we can create a fungal meroterpenoid production system. This method includes important benefits, in that we can exploit the novel biocatalysts by identifying the compounds accumulated in the heterologous expression system, and also re-engineer the biosynthetic pathway easily, by simply swapping one part of the biosynthetic machinery or feeding an un-natural substrate.

## 2. Pyripyropene A Biosynthetic Pathway

**Figure 3:**
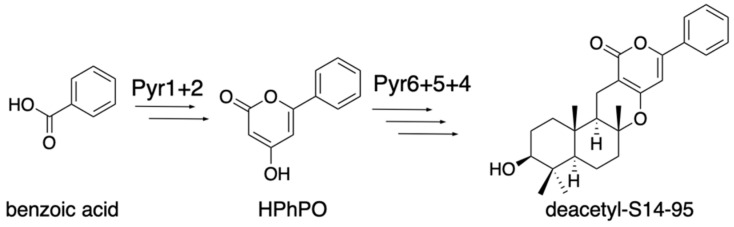
Un-natural product synthesis from benzoic acid with Pyr12456.

The earliest study of fungal meroterpenoid biosynthesis was the reconstitution of the early pyripyropene A biosynthetic pathway in the *Aspergillus oryzae* M-2-3 strain (Δ*argB*) [13]. The *pyr2* (polyketide synthase, PKS) gene was cloned into the pTAex3 vector [14], and the *pyr1* (CoA-ligase) gene was cloned into pPTRI [15], with the *amyB* promoter and terminator from pTAex3, to yield pTAex3-*pyr2* and pPTRI-*pyr1*, respectively. Similarly, *pyr5* (flavin-dependent monooxygenase, FMO) was cloned into pTAex3, and *pyr4* (transmembrane meroterpenoid cyclase, CYC) and *pyr6* (UbiA-type prenyltransferase, PT) were cloned into pPTRI with the *amyB* promoter and terminator, to yield pTAex3-*pyr5* and pPTRI-*pyr4*+*6*, respectively. Two sets of vectors, pTAex3-*pyr2* and pPTRI-*pyr1* and pTAex3-*pyr5* and pPTRI-*pyr4*+*6*, were introduced into *Aspergillus oryzae* M-2-3 [7]. The *A. oryzae*/*pyr1+2* was fed nicotinic acid and produced 4-hydroxy-6-(3-pyridinyl)-2H-pyran-2-one (HPPO), while *A. oryzae*/*pyr4+5+6* was fed HPPO and produced deacetylpyripyropene E. The structures of these products suggested that the following biosynthetic reactions, Pyr1 and Pyr2, produce HPPO from nicotinic acid, CoA, and malonyl-CoA; Pyr6 transfers the farnesyl group at C-3 to yield 3-farnesyl-HPPO; Pyr5 epoxidizes the terminal olefin of the farnesyl group; and Pyr4 facilitates the cyclization of the epoxidated prenyl chain via protonation to produce deacetylpyripyropene E (Figure 2). This was the first study that identified the function of the transmembrane meroterpenoid cyclase [16], and it paved the way toward investigations of the fungal meroterpenoid biosynthetic pathway. The *A. oryzae*/*pyr1+2* strain was fed benzoic acid and generated 4-hydroxy-6-phenyl-2H-pyran-2-one (HpHpO), which was then fed to *A. oryzae*/*pyr4+5+6* as a substrate to yield the un-natural meroterpenoid deacetyl-S14-95 (Figure 3) [13], illustrating the ease of mutasynthesis with this heterologous expression system.

## 3. 3,5-Dimethylorselinic Acid (DMOA)-Derived Meroterpenoid Biosynthetic Pathways

**Figure 4:**
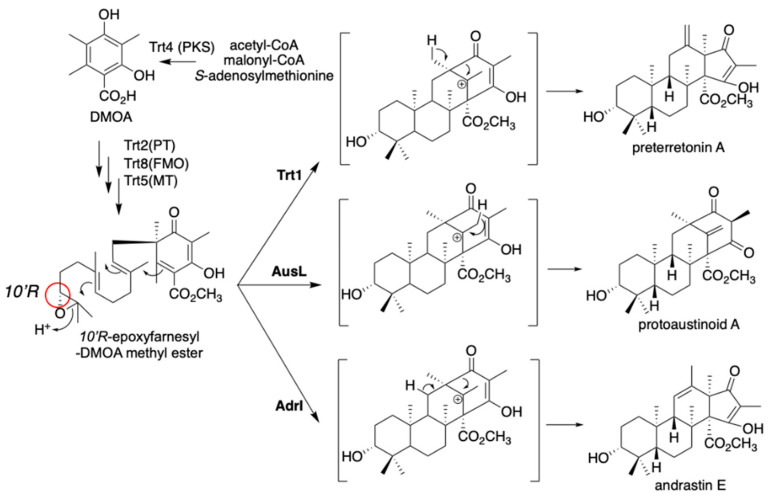
Diversification of DMOA-derived meroterpenoids with Trt1, AusL, and AdrI. C-10’*R* is highlighted with a red circle.

The fungal meroterpenoids derived from 3,5-dimethylorselinic acid (DMOA) exhibit structural diversity derived from various kinds of biosynthetic enzymes, including terpene cyclases and oxygenases [10,11,12]. In most of our expression studies, we employed *A. oryzae* NSAR1 [8] as the heterologous expression host. Since this strain possesses four auxotrophic mutations (Δ*argB*, *adeA*^-^, *sC*^-^, and *niaD*^-^), we can introduce four vectors harboring different auxotrophic markers, including *argB* (pTAex3) [14], *adeA* (pAdeA) [17], *sC* (pUSA) [18], and *niaD* (pUNA) [19]. By employing two vectors with the pyrithiamine resistance marker *ptrA* (pPTRI) [15] and the glufosinate resistance marker *bar* (pBAR) [20], we can introduce up to six vectors into a single expression host. Together, the strong *amyB* promoter on these vectors and the efficient metabolite efflux in the host facilitate the construction of a high-yield production system. By expressing the genes encoding Trt4 (PKS), Trt2 (PT), Trt8 (FMO), and Trt5 (methyltransferase) in *A. oryzae* NSAR1, we prepared a strain that produces 10’*R*-epoxyfarnesyl-3,5-DMOA methyl ester (Figure 4) [21,22]. With the additionally expressed *trt1* (CYC) in the *A. oryzae* system, the strain produced preterretonin A (Figure 4). When we swapped *trt1* with *ausL*, a homolog of *trt1* classified in the same clade of the phylogenetic tree, the *trt4285*+*ausL* transformant produced protoaustinoid A, a compound with a differently cyclized terpene ring (Figure 4). Furthermore, replacing *trt1* with *adrI* in the transformant also led to the production of another meroterpenoid, andrastin E [23]. These studies illustrate the usefulness of the heterologous gene expression system for metabolic engineering, in that we can create analogs by simply swapping one enzyme at a step in the biosynthetic reactions. A similar concept was also employed in the fungal indole diterpene combinatorial biosynthesis [24].

Recently, we produced novel meroterpenoids by introducing meroterpenoid cyclases from four different biosynthetic pathways into the *A. oryzae* host producing 10’*R*-epoxyfarnesyl-3,5-DMOA methyl ester, and obtained two novel mono-cyclized meroterpenoids [25]. It is intriguing that the four different meroterpenoid cyclases produced the same products from the non-native substrate.

**Figure 5:**
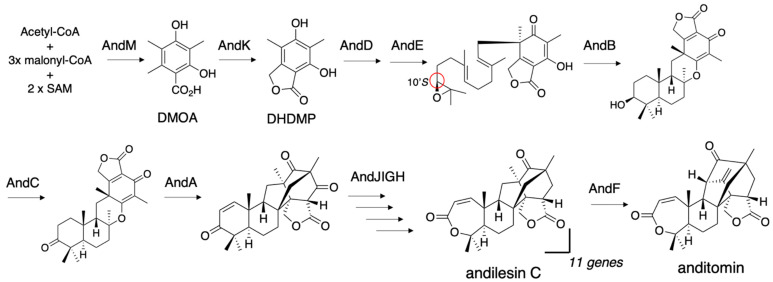
The 11-step anditomin biosynthetic pathway reconstructed in the *A. oryzae* heterologous expression system and the AndF reaction. C-10’*S* is highlighted with a red circle.

Anditomin is also a DMOA-derived meroterpenoid with the characteristic bicyclo[2.2.2]octane structure (Figure 5). In the anditomin biosynthetic study, we succeeded in the heterologous expression of 11 genes, *andM*(PKS)*+K*(P450 monooxygenase+hydrolase)*+D*(PT)*+E*(FMO)*+B*(CYC)*+C*(alcohol dehydrogenase)*+A*(α-ketoglutarate-dependent oxygenase)*+J*(Baeyer-Villiger monooxygenase)*+I*(ketoreductase)*+G*(acetyltransferase)*+H*(ene-reductase). To manage the number of vectors, two expression cassettes of PamyB-ORF-TamyB were connected with a linear vector by three-fragment in-fusion recombination [20]. First, we introduced pTAex3-andM, pUSA-andK+D, and pAdeA-andE+B into *A. oryzae* NSAR1, and then transformed the resultant strain with pUNA-andC+A and pPTRI-andJ+I to construct the nine-gene expression strain. Finally, pBARI-andG+H was transformed into the nine-gene transformant to yield the 11-gene expression strain, which produced andilesin C (Figure 5). pBARI was constructed by cloning the *bar* gene expression cassette with the *gpd* promoter from pBARGPE1 (FGSC) into pUC19. The transformants were selected with glufosinate, purified from a commercial herbicide [26]. To the best of our knowledge, the introduction of 11 genes into a single host is the largest number for *A. oryzae* heterologous expression. As we failed to introduce a 12th gene into the transformants, 11 genes might be the upper limit. The reaction of AndF was characterized through in vitro reaction by using andilesin C as a substrate. The characteristics of this biosynthesis are the generation of a 10*S*-epoxide, while the terretonin biosynthetic pathway generates a 10*R*-epoxide as a substrate for terpene cyclase, and the extensive terpene skeleton reconstruction by the α-ketoglutarate-dependent oxygenases AndA and AndF. The details of the catalysis by AndA have been extensively studied by a combination of X-ray crystal structural analysis and DFT calculations [27,28]. The generation of the 10*S*-epoxide in the *Aspergillus* expression system was also reported in the synthesis of the unique orthoester-bearing DMOA meroterpenoid novofumigatonin [29].

**Figure 6:**
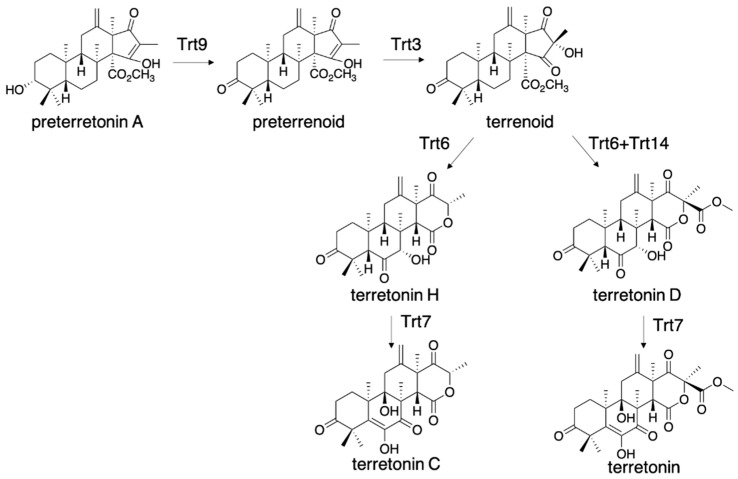
Biosynthesis of terretonin H and terretonin D from preterretonin A.

The D-ring of terretonin is highly oxygenated, and its biosynthesis is expected to include new biosynthetic enzymes. The expression of *trt42851+trt9* (alcohol dehydrogenase) *+trt3* (flavin monooxygenase) resulted in the production of terrenoid, which was further modified to terretonin H, with the additional expression of *trt6* (P450 monooxygenase) (Figure 6) [30]. The membrane fraction expressing Trt6 was also prepared from *A. oryzae/trt6* and used for the in vitro assay, indicating that *Aspergillus* gene expression system can also contribute to the preparation of membrane-bound enzymes that are only expressed in the eukaryotic host. Trt6 catalyzed C-7 hydroxylation, and the generated OH-7α formed a hydrogen bond with the carbonyl oxygen of the methyl ester. This hydrogen bond induces lactonization via the nucleophilic attack by OH-16 [30]. The resultant intermediate underwent retro-Claisen cleavage of the β-ketoester to the ring-expanded β-keto acid, and the following decarboxylation generated terretonin H. The additional expression of Trt14 (isomerase) in *A. oryzae*/*trt42851936* yielded terretonin D, which bears a methyl ester group, and facilitated the D-ring forming reaction. The molecular basis of Trt14 catalysis was reported in 2017, according to its crystal structure complexed with intermediates [31].

## 4. Ascochlorin/Ascofuranone Biosynthetic Pathways

**Figure 7:**
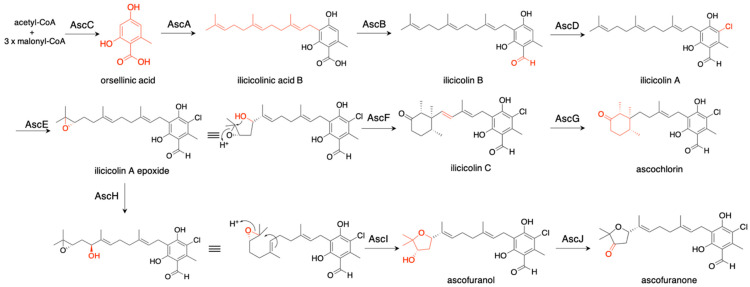
Biosynthetic pathways of ascochlorin and ascofuranone. The red fonts indicate the structural change after each biosynthetic reaction.

*Acremonium egyptiacum* produces two meroteropenoids, ascofuranone and ascochlorin, which are derived from the same intermediate, illicicolin A epoxide [32] (Figure 7). To investigate their biosynthetic pathways, we expressed *ascC* (polyketide synthase), *ascA* (prenyltransferase), and *ascB* (nonribosomal peptide synthetase-like reductase) in *A. oryzae* NSAR1, and the transformants produced illicicolin B with a yield of 0.71 mg/L. When we additionally expressed *ascD* (flavin-dependent halogenase), the transformant yielded a small amount of ilicicolin A (0.04 mg/L). The lower yield might be caused by the toxicity of the product against *Aspergillus*. *Aspergillus soja* was also employed as a heterologous expression host, and the proteins purified from this expression system were utilized for in vitro assays. The information from these experiments clarified the biosynthetic pathways of ascochlorin and ascofuranone, as shown in Figure 7. In ascochlorin biosynthesis, illicicolin A epoxide was protonated and cyclized into a (14*S*, 15*R*, 19*R*)-trimethylcyclohexanone ring structure by AscF to yield ilicicolin C, which was then oxidized by AscG (P450 oxidase) to produce ascochlorin. In ascofuranone biosynthesis, C-16 of illicicolin A epoxide was hydroxylated by AscH (P-450 oxygenase), and the product was cyclized into ascofuranol through 6-endo-tet cyclization by AscI. The ascofuranol was then oxidized by AscJ (NAD(P)-dependent dehydratase) to produce ascofuranone. AscI is a novel meroterpenoid cyclase sharing little similarity with known meroterpenoid cyclases, while AscF has similarity with Trt1 and known meroterpenoid cyclases [16,32,33]. The gene deletion of *ascF* resulted in the higher production of ascofuranone (501 mg/L) as compared with the wild-type strain (388 mg/L), illustrating the effectiveness of bioengineering to create a high-yield metabolite production system based on biosynthetic knowledge.

## 5. Diterpene Pyrone Biosynthetic Pathway

**Figure 8:**
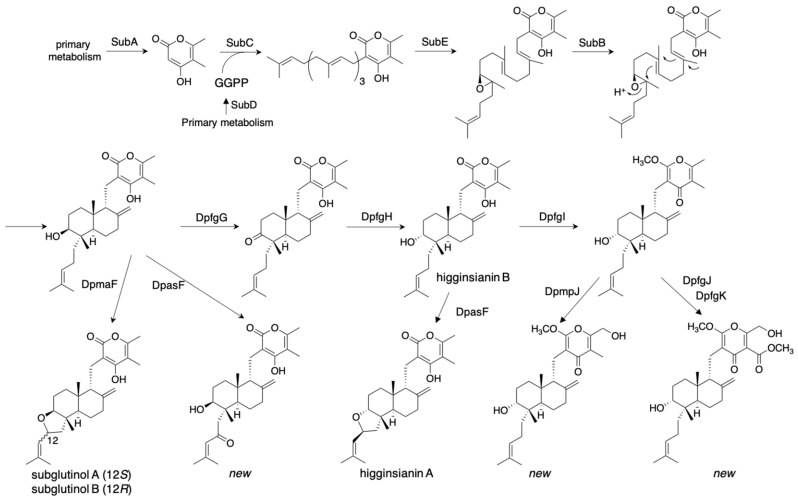
Combinatorial biosynthesis of diterpenoid pyrones in *A. oryzae*.

The most successful and thorough study on the combinatorial biosynthesis of fungal meroterpenoids was performed by Tsukada et al. [34]. They established ten biosynthetic pathways to yield 22 diterpene pyrones, including the immunosuppressive fungal meroterpenoid subglutinol A from *Fusarium graminearum* [35], and 15 novel analogs. They employed different combinations of the biosynthetic genes from five fungi isolated from a spider: *F. graminearum*, *Macrophomina phaseolina*, *Colletotrichum higginsianum*, *Metarhizium anisopliae*, and *Arthrinium sacchari*, in the *A. oryzae* NSAR1 heterologous expression system. The genes encoding SubA/DpasA (PKS), SubD/DpasD (geranylgeranylphosphate synthase), SubC/DpasC (PT), SubE/DpasE (FMO), and SubB/DpasB (CYC) from these five clusters produced the common diterpene pyrone intermediate with a high yield (87 mg/L). This intermediate structure was modified by the additional expression of DpmaF/DpasF/DpchF (flavin-dependent berberine bridge-like enzyme), to produce subglutinols A (89 mg/L) and B (6 mg/L) with their new analogs (Figure 8). Furthermore, they expressed DpfgG/DpmpG/DpchG and DpfgH/DpmpH/DpchH (NAD(P)-dependent reductases/dehydratases) to produce the 8-OH epimerized higginsianin B. DpfgI/DpmpI and DpfgK (methyltransferases), and DpmpJ and DpfgJ (P450 oxygenases) were employed to further diverge the pathway, which yielded a total of 22 diterpene pyrones (Figure 8). The well-designed cloning strategies enabled the authors to clone three or four gene expression cassettes into a single vector by two-step in-fusion cloning, and thus quickly construct diverse pathways. They reported a broad range of bioactivity tests, including anti-tumor, inhibition of insect innate immune system, anti-HIV, and anti-Alzheimer activities. Interestingly, the novel compounds produced via the un-natural pathways possess different bioactivities from the natural compounds. This study established the basis of synthetic biology to supply pharmacologically active compounds by heterologous expression systems.

## 6. Biosynthetic Pathways of Plant Meroterpenoids in *A. oryzae*

**Figure 9:**
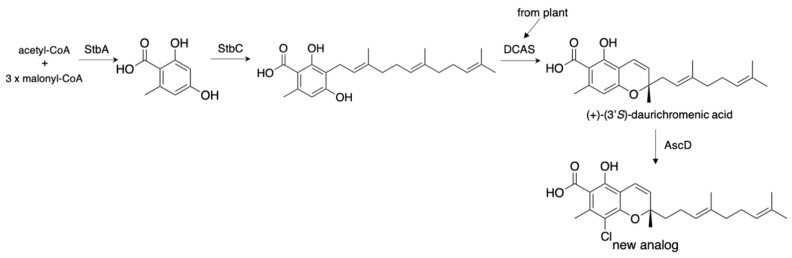
Production of plant meroterpenoids in *A. oryzae*.

In addition to the fungal meroterpenoids, plant meroterpenoids can be produced in *A. oryzae* by expressing the plant enzyme with fungal meroterpenoid biosynthetic enzymes. In 2017, we successfully constructed the plant anti-HIV meroterpenoid daurichromenic acid (1.23 mg/L) by expressing DCAS (flavin-dependent oxidase) from the plant, *Rhododendron dauricum*, with StbA (PKS) and StbC (PT) from *Stachybotrys bisbyi* (Figure 9) [36,37]. Furthermore, the additional expression of AscD from the *Fusarium* sp. [33] yielded its un-natural halogenated analogs (2.06 mg/L). These results illustrate the potential of the *Aspergillus* expression system to produce secondary metabolites. The recent successful syntheses of basidiomycete and plant terpenes in *A. oryzae* further encourage the production of secondary metabolites from other organisms besides filamentous fungi [38,39].

## 7. Concluding Remarks

*Aspergillus oryzae* is a powerful heterologous expression host for the production of polyketides and terpenoids. The high flux of its primary metabolism and the ease of genetic manipulation provide opportunities to produce various meroterpenoids. This powerful microbial platform will contribute to the generation of pharmaceutically important, un-natural molecules with relatively easy genetic and metabolic engineering. The recent development of a genetic manipulation system based on CRISPR-Cas9 in *A. oryzae* will further expand this versatile expression system, to produce high yields of diverse bioactive molecules, through engineering its primary metabolism and constructing multi-gene expression systems [40,41].

## Data Availability

Not applicable.
